# Delivering diabetes shared medical appointments in primary care: early and mid-program adaptations and implications for successful implementation

**DOI:** 10.1186/s12875-023-02006-8

**Published:** 2023-02-17

**Authors:** Andrea Nederveld, Phoutdavone Phimphasone-Brady, Dennis Gurfinkel, Jeanette A. Waxmonsky, Bethany M. Kwan, Jodi Summers Holtrop

**Affiliations:** 1 Department of Family Medicine, University of Colorado, Anschutz Medical Campus, Aurora, CO 80045 USA; 2 Department of Psychiatry, University of Colorado, Anschutz Medical Campus, Aurora, CO 80045 USA; 3 Adult and Child Center for Outcomes Research and Delivery Science, University of Colorado, Anschutz Medical Campus, Aurora, CO 80045 USA; 4 Deparment of Emergency Medicine, University of Colorado, Anschutz Medical Campus, Aurora, CO 80045 USA

**Keywords:** Diabetes, DSMES, Shared Medical Appointments, Adaptations, Implementation fidelity, Qualitative

## Abstract

**Background:**

Self-management is essential for good outcomes in type 2 diabetes and patients often benefit from self-management education. Shared medical appointments (SMAs) can increase self-efficacy for self management but are difficult for some primary care practices to implement. Understanding how practices adapt processes and delivery of SMAs for patients with type 2 diabetes may provide helpful strategies for other practices interested in implementing SMAs.

**Methods:**

The Invested in Diabetes study was a pragmatic cluster-randomized, comparative effectiveness trial designed to compare two different models of diabetes SMAs delivered in primary care. We used a multi-method approach guided by the FRAME to assess practices’ experience with implementation, including any planned and unplanned adaptations. Data sources included interviews, practice observations and field notes from practice facilitator check-ins.

**Results:**

Several findings were identified from the data: 1) Modifications and adaptations are common in implementation of SMAs, 2) while most adaptations were fidelity-consistent supporting the core components of the intervention conditions as designed, some were not, 3) Adaptations were perceived to be necessary to help SMAs meet patient and practice needs and overcome implementation challenges, and 4) Content changes in the sessions were often planned and enacted to better address the contextual circumstances such as patient needs and culture.

**Discussion:**

Implementing SMAs in primary care can be challenging and adaptations of both implementation processes and content and delivery of SMAS for patients with type 2 diabetes were common in the Invested in Diabetes study. Recognizing the need for adaptations based on practice context prior to implementation may help improve fit and success with SMAs, but care needs to be given to ensure that adaptations do not weaken the impact of the intervention. Practices may be able to assess what might need to adapted for them to be successful prior to implementation but likely will continue to adapt after implementation.

**Conclusion:**

Adaptations were common in the Invested in Diabetes study. Practices may benefit from understanding common challenges in implementing SMAs and adapting processes and delivery based on their own context.

**Trial registration:**

This trial is registered on clinicaltrials.gov under Trial number NCT03590041, posted 18/07/2018.

## Introduction

Self-management, including diet, exercise, use of medications, and stress management, is essential for good outcomes in type 2 diabetes. Patients often benefit from education and support for self-management, and guidelines recommend patient referral to self-management educational programs. Shared Medical Appointments (SMAs) are defined as clinical encounters in which a group of patients receives education and counseling, physical examination, and clinical support in a group setting [1]. Although SMAs have been shown to result in increased self-efficacy for patients with type 2 diabetes, increased patient and provider satisfaction [2, 3], and improved outcomes [4–8], most practices do not provide SMAs. This may be due to challenges with implementation. Barriers can include billing and reimbursement, lack of staff time and resources, work flow issues, and difficulty with patient recruitment [9, 10]. Thus, for primary care practices to be able to offer SMAs that have potential to improve patient health outcomes as well as increase satisfaction for both patients and practice members, implementation challenges must be overcome.

Implementing new programs invariably involves modifying interventions to fit practice context, considering factors such as staffing models and availability, payment structures, physical space, and patient populations and their preferences [11]. Understanding how complex interventions such as diabetes SMAs fit the primary care context may yield both improved implementation and sustainability. Some modifications are minor changes made to details such as scheduling or contacting participants. Others are considered adaptations, or modifications that are defined as “a process of thoughtful and deliberate alteration to the design or delivery of an intervention, with the goal of improving its fit or effectiveness in a given context” [12]. Examples of adaptations include changing curriculum materials for a low literacy audience or offering interventions to individuals outside of the target population. Some seemingly minor changes – such as changing sessions times from daytime to evening – can be adaptations if they are made strategically to address a key concern such as improving reach to participants. Adaptations can be fidelity-consistent, meaning not altering the core components of the intervention, or fidelity-inconsistent. Understanding how and why adaptations happen is important to understanding program delivery and effectiveness in diverse, real-world settings [13, 14].

Adaptations are often made by those implementing interventions in real-world care settings whether researchers studying these interventions want them to or not [15, 16]. Pragmatic trial design features explicitly allow for flexibility in intervention delivery – and thus offer an opportunity to study adaptations made by implementers. The Invested in Diabetes study was a pragmatic trial of two models of diabetes SMAs delivered in a range of primary care settings [17]. Guided by the Enhanced Replicating Effective Programs Framework, the research team made pre-implementation adaptations to the protocol and curriculum to enhance fit to participating practice contexts [18]. Additional adaptations made by practices (with or without the research team’s awareness) during early implementation were evaluated using multiple qualitative and quantitative data sources [19]. The purpose of the analysis presented here was to describe experience with SMA implementation and any adaptations made by practice staff. Specifically, we describe what was adapted, why it was adapted, when it was adapted, and how adaptations at the practice level were associated with fit, feasibility, reach, satisfaction, and outcomes to inform future implementation of diabetes SMAs in clinical care delivery settings.

## Methods

### Study design

The Invested in Diabetes study was a pragmatic cluster-randomized, comparative effectiveness trial designed to compare two different models of diabetes SMAs delivered in primary care [17]. Practices were randomly assigned to either a patient-driven or standardized diabetes SMA model condition. The Reach-Effectiveness-Adoption-Implementation-Maintenance (RE-AIM) [20] framework was used to guide evaluation of outcomes across conditions. To evaluate the RE-AIM implementation domain, we assessed practices’ experience with implementation, including any planned and unplanned adaptations at project baseline and about 9–12 months into implementation (termed mid-point).

#### Adaptation framework

We used the Framework for Reporting Adaptations and Modifications (FRAME) [12, 21] to report adaptations to SMA features common to both models and the features that were expected to differ between conditions. FRAME provides a classification system for the who, what, when, and where of adaptations, as well as the reasons for, goals of, relationship to core functions and whether adaptations are proactive or reactive. This structure provides a useful means to identify the characteristics (or components) of adaptations. Especially important to implementation are reasons for adaptation (grouped as increasing reach or engagement, increasing retention, improving feasibility, improving fit with recipient, addressing cultural factors, improving effectiveness, reducing cost, or increasing satisfaction) and what was adapted (grouped as program content, who is involved, recruitment, time devoted, follow up or tracking, scheduling, reimbursement, resources, and other). In addition, fidelity (to protocol and core elements) was tracked to ensure that evidence-based programs do not deviate substantially from what makes them efficacious, resulting in “voltage drop” or “program drift.” [11] For a full list of FRAME components and definitions as used here, as well as an in-depth description of our multi-method approach for evaluating adaptations, see Holtrop et al. 2022 [19]. The Colorado Multiple Institutional Review Board approved this protocol as expedited human subjects research. Verbal consent was obtained from interviewees as approved by our protocol. This trial is registered on clinicaltrials.gov under Trial number NCT03590041, posted July 18, 2018.

### Invested in diabetes SMAs

SMA features common to both patient-driven and standardized SMAs included use of a modified version of the Targeted Training in Illness Management (TTIM) curriculum [22, 23], an evidence-based group self-management education program for patients with type 2 diabetes. A trained facilitator (nurse, diabetes educator, or similar) used a TTIM instructor manual and patient handbook to deliver six 2-hour diabetes self-management education modules to groups of ideally 8–10 patients with type 2 diabetes [18]. Sessions were to be offered no more frequently than weekly and no less frequently than monthly. Medication management was provided as part of an associated and billable “prescribing provider visit” at each session by a treating clinician (physician, advanced practice provider, or clinical pharmacist were allowable).

Core components of the intervention driving fidelity considerations included inclusion of curriculum topic materials, prescribing provider visits, limiting the sessions to patients with type 2 diabetes and the condition specific components. The patient-driven SMA model differed from the standardized SMA model in that patient-driven sessions were delivered by a multi-disciplinary care team including a health educator, a behavioral health provider, and a diabetes peer mentor (versus the health educator alone in standardized). Also, patients in the patient-driven model selected the curriculum topic order and emphasis (vs a set order and prescribed time on each topic in standardized). Each practice agreed to recruit 36 or 72 patients and collect complete data on 30 or 60 patients with type 2 diabetes. The curriculum and patient materials were available in both English and Spanish.

### Setting and participants

Twenty-two primary care practices were randomly assigned to implement either patient-driven or standardized diabetes SMAs. Practices were provided with training on how to implement TTIM using their assigned SMA model type. Practice facilitators provided structured practice facilitation sessions during weekly to monthly check-ins. [17]

### Data sources

Table 1 summarizes the three sources of data used to evaluate adaptations and the time frame for data collection.

**Table 1.**
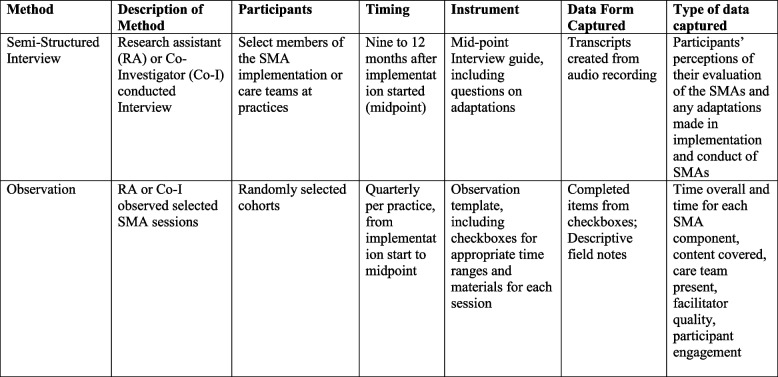
Data Collection Methods and Use for Studying Adaptations

^**a**^Reprinted from Holtrop JS, Gurfinkel D, Nederveld A, et al. Methods for capturing and analyzing adaptations: implications for implementation research. Implement Sci. Jul 29 2022;17(1):51. 10.1186/s13012-022-01218-3

### Data analysis

An experienced qualitative team was assembled that included the qualitative lead (JSH), physician researcher (AN), study manager (DG) and clinical health psychologist researcher (PPB).

The team conducted two main analyses. First, we conducted a traditional qualitative thematic analysis [24] with the midpoint interview data. The audio recordings were transcribed into text documents and then uploaded into ATLAS.ti (version 8, Scientific Software Development GmbH). The team identified codes using a collaborative process. One of the codes was “adaptation,” which was defined as any instance of the respondent noting a change from the intended curriculum or SMA process, whether explicitly stated in response to the question – “From when you started, did you make any changes to how you were conducting the sessions or the process?” (e.g., “Yes, we changed the prescribing provider from one of our physicians to the clinical pharmacist”) or inferred from knowing the protocol and noting that the explanation was different from the intended protocol (e.g., “We utilized our clinical pharmacist as the prescribing provider during the SMAs”). From this analysis of data specifically related to adaptions, a number of key themes related to adaptations, why they occurred, and how they were handled were identified. Second, facilitation notes and observation records were reviewed to capture adaptations discovered using those sources. All adaptations were cataloged according to FRAME, organized by FRAME components and reviewed for redundancies discussed within and between data sources. Qualitative findings reported in this paper were identified from interview analysis and were triangulated with data from observations and facilitator meeting notes.

## Results

Data reflected in this analysis were collected between January, 2019, and March, 2020, and consisted of 72 total transcripts of interviews with individual practice members, 33 observation templates and 168 practice facilitator notes (273 documents) from 21 practices (11 standardized vs 10 patient-driven; 12 Federally Qualified Health Center [FQHC] vs. 9 private practices). One practice was excluded because they withdrew from the study before completing mid-point data collection.

Four key findings were identified about adaptations practices made in response to the experiences in implementing SMAs in primary care. Broadly, the adaptations and challenges they reported related to either the process of implementation of SMAs in the practice, or content of the sessions and curriculum itself.

### Finding #1

Modifications and adaptations are common in implementation of SMAs.

We use the terms modifications and adaptations because sometimes we did not know the intent of the changes made to accurately state that these were always adaptations. We identified across practices 202 different modifications and adaptations; all practices reported at least three (range 3 – 22; mean of 9.6) from all data sources. Often the same modifications and adaptations were reported by different interviewees from within the same practice. More occurred during the implementation process (identifying staff to participate, training, developing workflows needed to coordinate and deliver SMAs) than in the intervention delivery (how the SMAs were actually conducted – what content was covered, who facilitated the sessions). Most of the modifications and adaptations across both implementation process and intervention delivery were unplanned/reactive with the stated or implied goal of improving the feasibility, reach (number of eligible patients that participated) or outcome of the SMAs. The majority stayed within the intervention protocol as stated by the study team, indicating fidelity to the core components of the intervention. Most were expected to improve the intervention fit to individual practice context, not to change the intervention itself; for example, changes to improve patient participation (e.g., offering incentives like meals or reward items) or scheduling (e.g., offering sessions at a variety of times), rather than to program components. Changing or adding different patient recruitment strategies due to difficulties with recruitment was common for all practices, and a universal challenge. Scheduling changes were frequently made to better fit patient schedules (e.g., sessions later in the evening or on weekends) and practice staff availabilities (e.g., running concurrent cohorts back to back instead of on several days). However, notable examples of modifications and adaptations to the program were found in interviews and facilitator notes. For example, prescribing provider visits were often moved to occur before or after the session due to issues such as patients missing content or reduced in frequency due to effects on provider productivity.

### Finding #2

While most adaptations were fidelity-consistent supporting the core components of the intervention conditions as designed, some were not.

While most adaptations were within protocol, there were notable examples of adaptations that broke from the core components, primarily involving patient recruitment, personnel involvement, and content covered. Interviews and facilitator notes detailed adaptations such as including patients with type 1 or pre-diabetes, often because the Invested sessions were the only available diabetes education at the clinic. Observations showed that the care team members delivering the assigned SMA model were at times inconsistent with those expected per condition protocol. For instance, the peer mentor was often absent in the patient-driven condition, or a behavioral health provider was present in the standardized condition. This was corroborated in interviews by several practices stating the logistical difficulties of coordinating with peer mentors to attend the SMA. Observations also showed the exclusion of key portions of the curriculum, and more often, inadequate time spent on the topics and discussion. Interviews and facilitator notes discussed additions or subtractions to the curriculum due to cultural adaptations or the facilitator’s comfort with the material. In addition, some adaptations were made to bring implementation back to being fidelity consistent; for example, removing content from presentations that was not part of the curriculum.

### Finding #3

Adaptations were perceived to be necessary to help SMAs meet patient and practice needs and overcome implementation challenges. Table 2 lists implementation challenges with an illustrative quote and practice adaptations to address challenges.

**Table 2:** Implementation challenges and modifications/adaptations discussed or observed

Implementation Challenge		Modifications and Adaptations made to improve implementation	Illustrative quote, role of team member, type and study number of practice
Patient-level challenges	Recruitment – Patient Identification	Be flexible on who participates Clinicians talk to patients directly rather than relying on other staff to recruit patients Use personal connections or interactions to recruit patients to SMAs	“The very first cohort, we had only the very high A1Cs. The second cohort, our staff wanted to involve patients with lower A1Cs so that they could sort of lend a perspective to those with high A1Cs, but this did not result in any more patients being engaged in showing up. For the third cohort, we just opened it up so that we just had a larger pool to choose from.” (provider, Private Practice # 11) “We do have patients who are pre-diabetic that do come to the groups. I’ve gotten those referrals from the clinic providers, specifically, actually. One of our clinics, they just have a large pre-diabetic population there, and they send quite a few referrals over” (coordinator, Federally Qualified Health Center # 04) “I think the first cohort…I went down the list of people and said, “These ones might be potential candidates, just ‘cause I know them or they’re people who are reliable, generally.” Then the second go-around was more, “Oh, yeah, this person would be good. I talked to them today. They expressed interest.” (provider, Federally Qualified Health Center # 03) "If I have a patient that I see that I think might be a good fit, I’ll recruit them to the class.” (coordinator, Private practice #07)
	Recruitment – contacting and enticing patients	Make it sound like something patients don’t want to refuse Appeal to what is happening outside the clinic Emphasize that the patient’s primary care provider thinks they could benefit Recognize that recruitment is challenging	“On the left hand side it says, "What is a diabetes shared medical appointment?" and the other side lookin’ just like a birthday invitation…They get this invitation and they call me, their buttons are already pushed. They wanna come.”(health educator, Federally Qualified Health Center # 05) “It’s our biggest class, and it was very effective when I said, “You know, the holidays are coming, and one of the things we’re gonna cover is how to deal with stress and the pressure of the holiday season and all the meals and everything.” (coordinator, Private practice #04) “I do the cold calls. I’ll just go down the list, and I’ll start calling them…it’s really effective if I say, “Well, Dr. so-and-so thought you might be a good candidate for this class and wanted me to give you a call.” (coordinator, Private practice #04) “We’ve tried a couple of different things. At first, we were doing a lot of cold calls on top of just providers referring. The cold calls have been very hard pulling the data from the EHR. Very, very few get back to us. After 60 phone calls you get maybe 5…Or those patients no-show when they sign up for the group cause you got them on the phone and they felt bad.” (health educator, Federally Qualified Health Center #06)
	Retention	Recognize that there will be no-shows and drop-outs and plan for that	“Every time, we have multiple patients who say they’re interested, but then they drop out for a variety of reasons. I think this time, they called 20, 25 people…but still, this cohort’s only consisting of one to two consistent people.” (provider, Private practice #11)
Practice-level challenges	Physical Space	Modify or find other space to accommodate the program if it isn’t working	“It used to be downstairs, but we had two really tiny rooms. If we have more than seven it’s super crowded. We moved upstairs to a really big room, but then that leaves where does the provider see the patient?” (health educator, Federally Qualified Health Center #06) Yeah, so now, we use the hospital conference room space, which actually has been really nice. They have classroom-style spaces and so I think that’s actually been better than a conference room space (coordinator, Federally Qualified Health Center #04)
	Provider involvement and efficacy	Titrate the provider visit portion according to the participation and timing	“I think, a hard sell to get more for—to lose, I think, the regular patient care for more than two sessions. I think that was—I think that was the the biggest thing. I think there’s a lot of concerns on the side of how many patients we can see, that need to be seen on this side of town, that have a need for regular primary care.” (provider, Federally Qualified Health Center #04) “Honestly, my nurse practitioner and I have felt like they’re not super helpful. That’s part of how we need to do this program, in order to get paid. Unfortunately, that’s the only way for insurance cover the group visits at this point, is to do that face-to-face 10-min visit with the patient.”(provider, Private practice #06) “If an MD is taking 2-plus hours out their schedule, then they need to see 10 or 12 patients to make that worth their time. If there aren’t 10 or 12 patients to see, then it’s just not worth their time from a productivity model” (coordinator, Private practice #07–10)
	Staff turnover	Develop a strategy for addressing employee turnover and retraining	“We have had some turnover in the MAs [who] were helping us with group, the medical assistants. We’ve had to bring on whoever is the new MA who was working with us up to speed on what happened in the diabetes group.” (provider, Federally Qualified Health Center #05)
	Scheduling	Base SMA timing on patient characteristics (e.g. working vs. retired) Consider the reality of space and staffing considerations and how they align Be prepared to make decisions throughout the process	“Honestly, I think the hardest part was the scheduling piece, because it just seemed like in terms of the patients that we got for different times—’cause we did a few lunchtime groups, and then we did a 4:00 to 6:00 p.m. group.” (BHP, Private practice #06) “We have a lot of providers who just aren’t available at the same time during the week or have other commitments when the room was available. Our practice has outgrown our space as it is, and so finding a space to have these groups that is patient-friendly and comfortable has limited when we can have the visits.” (provider, Private practice #11) “Because there was just too much would happen in a month—the provider part got too comprehensive. We thought if we change it to weekly, there’s less that can happen in a week.” (health educator/coordinator, Federally Qualified Health Center #03)

### Patient-level challenges

#### Recruitment – patient identification and contacting and enticing patients

Most practices tried several different strategies to identify, connect with, and interest patients in attending SMAs, from recruiting only from a specific provider’s panel, recruiting from registries of patients with diabetes, advertising in waiting rooms and exam rooms, and having staff or providers recommend SMAs to patients with diabetes. Often times, practices ended up adapting their strategy to better fit their workflow and added additional strategies to help bolster patient interest, including making special invitations or marketing as a support group. Many started by asking providers to identify patients they thought would be interested but then transitioned to using registries from electronic health records to identify patients with diabetes, often starting with patients with high hemoglobin A1C values. Most practices eventually opened the groups to any patient with type 2 diabetes. As noted in theme 2, some practices opened recruitment to patients with type 1 or pre-diabetes due to limited available resources for diabetes education.

Despite all of these adaptations and strategies, recruitment was still challenging for many practices, particularly because this was a study with specific recruitment goals. However, most interviewees reported that there were interested patients in their practices and identified motivation to improve self-management as the key patient characteristic that predicted successful recruitment to attend SMAs.

#### Retention

Another challenge was retaining patients once they joined an SMA cohort. Practices described patients dropping off after a few sessions and therefore tried different approaches to maintain patient interest. For example, practices offered incentives or provided meals during sessions to encourage people to attend (this is recommended in the TTIM training materials). Practices also reported changing the schedule of provider visits to limit co-pays, as patients sometimes objected to paying a co-pay for six visits and dropped out for this reason.

### Practice-level challenges

#### Physical space

Many practices realized after starting SMAs that they did not have an ideal physical space for group visits. To adapt, they found other areas to conduct the sessions such as break rooms and conferences rooms normally used for staff meetings or waiting rooms if SMAs were offered outside of normal clinic hours. One practice described having to reserve a space outside of their clinic at the adjacent hospital to accommodate their groups.

#### Provider involvement and efficacy

Many practices modified and/or adapted the prescribing provider visits. Many practices identified the educational component as more valuable, while the prescribing provider visits were seen as disruptive and modified them so they occurred before or after the SMA. Other modifications included having a set provider conduct all visits for a SMA cohort rather than having every person see their primary care provider or changing the personnel type who did the visits from physicians to clinical pharmacists. Other changes were true adaptations, including limiting the total number of provider visits per cohort either systematically (i.e., having patients see a provider at session 2 and 6) or by patient choice (i.e., always offering a concurrent provider visit but not requiring it because as noted above, a weekly co-pay associated with provider visits at each session affected retention). Some had initially envisioned having the provider attend all groups and see each patient every time, but found that reimbursement for these visits did not cover missed clinic time by the provider. At the same time, it was recognized that with current billing structures, provider visits are the most sustainable way to be reimbursed for these sessions, and therefore many practices considered provider visits necessary. Including provider visits differentiates SMAs from group education sessions; therefore, not including them was a considered a major adaptation as without provider visits, the sessions become diabetes self-management education. Decisions to reduce or eliminate these visits were discussed extensively with the practice facilitators and permission to do so was often sought from the study team. As a result, the study team determined that practices could have each patient have a prescribing provider visit at a minimum of 1 SMA session and still consider the intervention fidelity-consistent.

#### Staff turnover

Staff turnover was a challenge for practices as well, particularly if a practice champion left the practice or couldn’t participate in the conduct of the visits due to time or other constraints. This frequently resulted in sessions being facilitated by new staff who hadn’t been adequately trained or disruption in scheduled cohorts, especially when there was no plan to onboard new staff or available back up personnel. Interviewees discussed the need to have a practice champion for the SMAs to run successfully, and the need to replace them quickly if they left.

#### Scheduling

Practices also experimented with SMA scheduling. Many changed the interval between sessions and time of day they offered the SMAs. For example, some practices started with monthly visits and found that weekly or biweekly visits worked better for either patients, personnel, or both. These changes were primarily made because of patient acceptability, but also due to provider schedules or facility issues. Flexibility in scheduling SMAs, particularly for after-hours times, was very beneficial but not always feasible.

### Finding #4

Content changes in the sessions were often planned and enacted to better address the contextual circumstances such as patient needs and culture.

Changes to the content and actual delivery of the materials in the sessions were often discussed during interviews and also were identified during observations. The challenges practices experienced in delivery and related adaptations and successful strategies are summarized with illustrative quotes in Table 3.

**Table 3:** Challenges and resulting adaptations around intervention content and delivery

Challenge Identified	Strategies and Adaptations done to assist implementation	Illustrative quote, role of team member, type and study number of practice
Content perceived to be not suited for patient population	Added or adjusted program content	“I know with the Spanish one she does a lot more visuals because the patients don’t always read all the stuff. For the IDEA approach she printed out a light bulb, and doing more visuals to help with the words that they don’t always read. I think that’s been a little bit helpful.” (health educator, Federally Qualified Health Center #06)
Content difficult to deliver according to program plan/timing	Remove or change timing of content delivery	“I did do the medication one in the first cohort, and it just felt so pressured. One of the patients actually told me she felt overwhelmed. It just seemed like the most practical piece to remove and still have the meat of that whole section in that curriculum.” (BHP, Federally Qualified Health Center #04)
Disagreement with nutrition information/content	Alter or add to program content	“It did seem the carb counting piece is something that we’re just not emphasizing that anymore. They immediately think carbohydrates are the bad guy and remove that from their diet and eat summer sausage and cheese cause that’s the message when you’re carb counting, so therefore carbs must be bad…I also think that class was the longest one and you had one patient comment that they just had so many more questions. They wanted sample meal and snack ideas, some real examples.” (health educator, Private practice #06)
Content difficult for patients with low literacy levels	Added visuals	“Like I mentioned, there’s some patients that didn’t understand the wordy part of some of our things, and so it was just really improvising and trying to show them a way of visually being able to see ‘em” (health educator, Federally Qualified Health Center # 08)
Mental health content not seen as appropriate for patient population	Removed specific content	“I get a little frustrated with the curriculum sometimes. With the curriculum we’re using now, there are components of it where I don’t feel very proficient because they’re so behavior change, mental health focused”(health educator, Federally Qualified Health Center #05) “The only one that we don’t present too much anymore is the one with the severe mental health problems. It’s going down too deep for a six visit group. Goin’ into schizophrenia meds and stuff like that with these folks…That’s a little too much.” (health educator, Federally Qualified Health Center #05)

Despite study protocol requiring the use of the established TTIM curriculum including the facilitator’s manual with scripts, PowerPoint slides, and patient handout materials, many practices adjusted program content. The patient-driven practices were told this was acceptable in advance as this SMA model was meant to be more responsive to patient needs; this change was thus fidelity-consistent for the patient-driven condition. Standardized practices were asked not to change the curriculum. These adjustments reflected a desire to make the content better suited to their patient populations, for example, making it more culturally appropriate for native Spanish speakers or using pictures for patients with low literacy levels. In general, these curriculum changes were modifications or cultural additions discussed with the implementation facilitators before the practices made the changes or were revealed during interviews. Some staff also described adjusting the content if they didn’t agree with points made or feel comfortable with the content. Health educators also added nutrition content and frequently commented on the abundance of patient questions related to nutrition they received that were not adequately addressed by the TTIM curriculum. In addition, as program facilitators became more comfortable with the content, they reported less verbatim teaching. They reported adding their own visuals or PowerPoint presentations, sometimes shortening the sessions or not covering everything in the curriculum. This was especially true for the modules related to mental health, stress, and coping, primarily the serious mental illness; however, practices were given a choice of two options for behavioral health delivery, which was not an adaptation.

## Discussion

We found that primary care practices participating in the Invested in Diabetes study and implementing an evidence-based curriculum to provide SMAs to patients with type 2 diabetes made multiple modifications and adaptations during this process, most commonly to improve patient recruitment and to improve feasibility for the practice staff. The majority were made in alignment with the curriculum and with fidelity to the study protocol for their assigned SMA conditions. However, a sizable minority of changes were not fidelity-consistent, and these adaptations generally related to the delivery of the curriculum (time spent on topics or additional visuals/tool), including patients with diagnoses other than type 2 diabetes or practice staff roles (omitting prescribing providers, having a behavioral health provider present in the standardized condition; not having a peer mentor in the patient driven condition) to accommodate practice circumstances and to enhance the effectiveness of the program. Although not the subject of this paper, a separate analysis of these changes on the fidelity to each condition has been undertaken in examining the effect of each treatment arm on outcomes. Our main finding is that adaptations occurred in both conditions and were common.

The literature suggests that clinicians and practice teams value adaptability in SMA protocols and approaches, and that interventions must be adaptable in order to be widely implemented in primary care [25]. However, studies have shown that curriculum fidelity to evidence-based interventions is a crucial factor in improving targeted outcomes [26]. Finding the balance between these considerations is likely the key to successful implementation and sustainment of evidence-based practices in primary care. Indeed, this is the motivation for considering context and balancing fidelity with adaptation. These results support that successful implementation of new interventions, such as the SMAs for diabetes in this study, requires a balance between remaining flexible and supporting adaptations to individual practice context and strict intervention fidelity.

In addition, these results make clear that modifications and adaptations are common and on-going, as many of occurred well after pre-implementation planning and occurred often and at all sites throughout implementation. Encouraging practice teams who are implementing new interventions to expect to modify due to context may increase the likelihood that interventions will be successful initially. Modifications and adaptations are also necessary for sustainment of interventions as practice context changes over time. Understanding what core components make evidence-based programs successful, and identifying and limiting adaptations to those components, could ensure greater health outcomes seen overall.

Some of the challenges associated with delivering diabetes SMAs in Invested stemmed from the research protocol rather than the curriculum or the SMA model itself. Struggles with patient recruitment – and the associated stress – may have been due to the fact that practices had been asked to recruit a certain number of patients to participate so that the research objectives would be met.

SMAs have been shown to be effective in type 2 diabetes and in general for management of chronic disease. However, implementation in primary care can be challenging, and many practices may feel that these challenges are unique to them or find them too frustrating to continue [9, 27]. Knowing what challenges are likely and then having a plan to address them could be useful for others. Understanding which issues related to practice context are most challenging will help practices interested in implementing SMAs to plan well and consider their own context. Also, understanding which adaptations may affect fidelity is important for research and evaluation of SMAs programs. Practice implementers of SMAs should particularly take note of challenges and adaptations described in this paper, which show major issues with implementation factors at the patient and practice level, notably recruitment, scheduling, and personnel/provider involvement, as well as issues in content delivery.

Results from this study lead to the following recommendations for practice teams considering implementing diabetes or other SMAs:

1. Expect to make changes at all stages of the implementation. Before implementation, consider your patient population, resources and circumstances. How will the content fit your patients? Are there cultural changes? Literacy issues? How will the number and timing of sessions fit what the patients will accommodate? Will additions or subtractions from selected curriculum affect its presumed efficacy?
2. Start with what you think will work and then tweak it according to what you learn. Much like quality improvement, pay attention to what is working and make changes in response to unexpected events (e.g., staff turnover) and because something is just not working well (e.g., reaching patients through a particular recruitment strategy). Continuing to do something that is not working is not going to be useful. However, also pay attention to what is really the critical “intervention” that is helping to facilitate change in the patients. For example, if there is a curriculum that is being followed, take care to not make too many or major changes to the content and methods as these are likely to deliver key intervention components that relate to effectiveness for patients.
3. Be prepared for challenges related to specific parts of implementation. We identified several common pieces of the implementation process and conduct of SMAs that were adapted across practices in this study. Our participants found success with the strategies outlined in Tables 2 and 3 and these could be starting points for future SMA implementation projects.

### Limitations

The results of this study should be interpreted with caution as they may not represent all practices or circumstances. These data were provided from qualitative information gathered from practices in two states participating in a study of diabetes SMAs. Therefore, these data may not represent other practices in other geographic areas and circumstances. Although all interview candidates were asked the same questions about adaptations both in the implementation process and in the SMAs themselves, it is possible that not all adaptations were discussed in the interviews. In addition, we conducted these interviews prior to the COVID-19 pandemic, so did not capture any adaptations related to challenges practices likely faced secondary to the pandemic such as implementation of virtual visits. We chose to use the FRAME to categorize our responses generally, but did need to make some modifications to fit our study, which may not allow for comparisons across other evaluations utilizing FRAME but also illustrates the importance of adaptability in academic implementation frameworks.

## Conclusion

SMAs for patients with diabetes can be beneficial for improving diabetes clinical and self-management outcomes. Implementation of SMAs can be a challenge, and adaptations are common, sometimes are fidelity-consistent and other times are not, often relate to practice-level context or are related to patient population factors. These adaptions can contribute to practice success in implementing the intervention. Practice teams may benefit from understanding common barriers to implementation of SMAs and ways they can adapt a curriculum or approach to better fit their context and patients.

## Data Availability

The datasets used and/or analysed during the current study are available from the corresponding author on reasonable request.

## Abbreviations

SMA: Shared Medical Appointment
RE-AIM: The Reach-Effectiveness-Adoption-Implementation-Maintenance [framework]
FRAME: Framework for Reporting Adaptations and Modifications
TTIM: Targeted Training in Illness Management
RA: Research Assistant
Co-I: Co-Investigator
FQHC: Federally-qualified health center
